# Heavy Metal Pollution and Risk Assessment of Sediments in Liuye Lake Based on Monte Carlo Simulation

**DOI:** 10.3390/toxics14040298

**Published:** 2026-03-29

**Authors:** Gao Li, Zhen Xu, Jie Zheng, Yuheng Xie, Lixiang Li, Yi Peng, Kun Luo, Yang Liu

**Affiliations:** Changsha General Survey of Natural Resources Center, China Geological Survey, Ningxiang 410600, China; ligao20226@163.com (G.L.); xuzhen@mail.cgs.gov.cn (Z.X.); zhengjie@mail.cgs.gov.cn (J.Z.); xieyuheng@mail.cgs.gov.cn (Y.X.); pengy@mail.cgs.gov.cn (Y.P.); luotoday@163.com (K.L.); liuyang03@mail.cgs.gov.cn (Y.L.)

**Keywords:** Liuye lake, surface sediments, heavy metals, Monte Carlo simulation, ecological risk assessment, human health risk assessment

## Abstract

Heavy metals in lake sediments represent typical persistent contaminants characterized by recalcitrance, bioaccumulation potential, and delayed toxic effects, thereby exerting sustained adverse impacts on lacustrine ecosystem stability and human health. Liuye Lake is a representative small-to-medium urban lake impacted by ambient domestic sewage discharge and agricultural non-point source pollution, with documented nitrogen and phosphorus enrichment. However, the contamination profile of heavy metals in its surface sediments has not been systematically investigated to date. In this work, surface sediment samples were collected from Liuye Lake, and nine heavy metal elements (As, Cd, Cr, Cu, Hg, Mn, Ni, Pb, Zn) were determined. An integrated approach incorporating Monte Carlo simulation, the geo-accumulation index (*I_geo_*), and the enrichment factor (*EF*) method was employed to assess the ecological risk and human health risk imposed by these metals. The results revealed the following: (1) Average concentrations of eight heavy metals exceeded the background values of the Dongting Lake water system, with the exception of As, and Hg displayed potential localized anomalies. (2) Surface sediments were collectively categorized as slightly contaminated, with Hg identified as the primary pollutant, followed by minor contamination of Mn, Cr, and Ni; Monte Carlo simulation further suggested a probable risk that Mn contamination could progress to moderate levels. (3) All heavy metals posed low potential ecological risk, with an overall potential ecological risk index (*RI*) of 62.71, where Cd, Hg, and As were the dominant contributors. (4) Both non-carcinogenic and carcinogenic risks were generally within acceptable limits, whereas children exhibited higher non-carcinogenic susceptibility relative to adults; As and Mn were the leading contributors to non-carcinogenic risk, while Cr and As dominated carcinogenic risk. This study offers a scientific foundation for the prevention and control of heavy metal pollution and the ecological management of urban lakes.

## 1. Introduction

As essential components of terrestrial ecosystems, lakes deliver core ecological services including regional climate regulation, biodiversity maintenance, and freshwater supply, while also acting as important sinks and sources for diverse pollutants in watershed systems [1,2]. Of particular concern are heavy metals in lake sediments, which are typical persistent environmental pollutants. These elements enter lacustrine systems via natural processes or anthropogenic activities, and eventually become concentrated in sediments through physical sedimentation, chemical adsorption, and biological enrichment [3,4]. Heavy metals are distinguished by their non-biodegradable nature, strong bioaccumulation potential, and delayed toxic effects. Even at low ambient concentrations in water, they can steadily accumulate in sediments, generating persistent contamination risks and long-term threats to lake ecosystem stability and human health [5,6]. Driven by rapid industrialization, urbanization, and agricultural modernization, human activities have become the dominant source of heavy metals in lake sediments [7,8]. Major exogenous inputs include industrial mining and smelting, agricultural fertilization, sewage irrigation, landfill leachate, and vehicle emissions. These contaminants reach lakes via direct wastewater discharge, atmospheric deposition, and surface runoff [9,10,11]. Upon interacting with suspended particulate matter in the water column, heavy metals gradually settle and form contaminated layers at the lake bottom through long-term deposition, thereby posing considerable ecological risks [12]. Growing evidence from recent investigations [13,14,15,16,17] confirms that heavy metal pollution in lake sediments has evolved into a widespread and prominent environmental issue worldwide. Extensive and in-depth studies conducted by global researchers have produced abundant results covering various lake types and geographical regions.

Zilkey et al. [18] evaluated heavy metal pollution in sediments from 167 lakes across Canada, and reported that roughly 70% of sampling sites failed to meet sediment quality criteria for at least one toxic element, with higher contamination levels closely related to urbanization and low topographic relief. Gabriele et al. [19] explored how historical mining and electronic waste recycling affected the spatial patterns of trace toxic elements (TCEs) and rare earth elements (REEs) in boreal lakes of Canada. Their results indicated that lake zones adjacent to smelters showed significantly elevated concentrations of multiple metals in both water and sediments, and Zn, Cr, and Pb contents in fish muscle tissue exceeded permissible safety limits. Perrotta et al. [20] analyzed the migration and transfer of copper in aquatic–riparian food webs affected by legacy copper mining activities. They detected extremely high copper concentrations in sediments of Torch Lake, Michigan, USA, confirming that metal pollution could cross ecosystem boundaries via trophic transfer from aquatic insects to riparian insectivorous spiders. Ben Saad et al. [21] examined the spatiotemporal dynamics and source apportionment of heavy metals in lagoon sediments within World Heritage Sites. They observed excessive accumulation of Cr, Pb, Zn and other metals, mainly derived from industrial districts and ports, with pollution intensity gradually declining from Lake Bizerte to Ichkeul Lake. In China, studies regarding heavy metals in lake sediments have mainly concentrated on large lakes located in economically developed regions, such as the middle and lower Yangtze River basin and the Pearl River Delta, including Poyang Lake, Dongting Lake, Taihu Lake and Chaohu Lake [22]. As a large shallow lake on the eastern plain of China, Taihu Lake is under intense pressure from industrial, agricultural and urban activities, making it a key research area for sediment heavy metal studies in China [23]. Researchers systematically collected sediment cores and surface samples from typical regions of Taihu Lake, including Meiliang Bay, Gonghu Bay and East Taihu Lake, and determined the concentrations and spatial distribution of As, Cd, Cr, Cu, Pb, Zn and other heavy metals. The results demonstrated that sediment heavy metal contents in Meiliang Bay, which suffers from severe industrial pollution, were markedly higher than those in other regions. Among these metals, Cd and Hg posed relatively high ecological risks and thus demanded priority monitoring [23]. As an important regulatory reservoir in the Yangtze River basin, research on Dongting Lake has mostly focused on agricultural non-point source pollution and the responses of wetland ecosystems to sediment-bound heavy metals. Studies on the uptake and accumulation of heavy metals by wetland plants showed that species such as Phragmites australis and Typha angustifolia present certain purification capacities, offering theoretical support for wetland ecological restoration in Dongting Lake [24]. As the largest freshwater lake in China, investigations on Poyang Lake have primarily focused on the temporal accumulation characteristics of sediment heavy metals and the impacts of hydrological conditions on metal migration and transformation. Analyses of sediment core records from different periods revealed temporal variation trends of heavy metal concentrations, with Cr, Zn and other metals showing increasing trends in recent years driven by the development of industry and agriculture around the lake [25]. Researchers across the globe have established a series of approaches to assess heavy metal contamination, including the single-factor pollution index method [26] and the Nemerow comprehensive pollution index method [27]. Based on these fundamental methods, several typical ecological risk assessment models have been widely applied, such as the geo-accumulation index method [28], the enrichment factor method [29], and the potential ecological risk index method [30]. Furthermore, human health risk assessments have been implemented by combining heavy metal concentrations with multiple exposure pathways.

Although considerable progress has been made in the study of heavy metals in lake sediments, several limitations still exist in current research. First, the geographical distribution of relevant studies remains highly unbalanced. Previous studies have mostly concentrated on large lakes in economically developed areas and suburban lakes suffering from intensive industrial and agricultural disturbances. By contrast, research on small and medium-sized urban lakes, which are closely related to daily human life, is still insufficient. As key components of urban ecosystems, these lakes directly affect the living environment and health of urban residents. Meanwhile, their fragile ecological structure and weak environmental capacity make them extremely sensitive to external pollution. Owing to the lack of systematic investigation, the pollution characteristics, source apportionment, and potential risks of heavy metals in these lakes remain unclear, which restricts the formulation of scientific remediation and protection strategies [31,32]. Second, in terms of human health risk assessment, exclusive dependence on models recommended by the United States Environmental Protection Agency (USEPA) may reduce the reliability of evaluation results, due to differences in individual parameters such as age, physical condition, gender, and metabolic rate. The health risk models recommended by USEPA usually adopt fixed exposure parameters based on population averages, which cannot reflect the heterogeneity among different groups such as children, adult males, and adult females. In practice, exposure levels and sensitivity to heavy metals vary widely among individuals. The use of fixed-parameter models ignores individual differences, leading to deviations between assessment results and actual risks. This weakens the scientificity and credibility of health risk evaluations, making it difficult to provide accurate decision support for the health protection of diverse populations [33,34].

To address the shortcomings in current research, this study investigates heavy metal pollution in surface sediments of Liuye Lake, hailed as “China’s premier urban lake.” As a typical small-to-medium-sized urban lake, Liuye Lake is closely intertwined with the lives of city residents. Its ecological and environmental quality significantly impacts residents’ quality of life and health safety. Previous studies indicate that while Liuye Lake generally maintains good water quality, it faces moderate nutrient pollution from surrounding urban sewage and agricultural nonpoint sources, posing a risk of eutrophication [35]. However, no studies have reported on heavy metals in the surface sediments of Liuye Lake, leaving the pollution status, sources, and potential risks of heavy metals in these sediments unknown. This limitation hinders a comprehensive understanding and scientific management of the lake’s ecological environment quality. Therefore, this study collected surface sediment samples from various regions of Liuye Lake and analyzed nine heavy metal indicators—As, Cd, Cr, Cu, Hg, Mn, Ni, Pb, and Zn—to determine heavy metal concentrations and the severity of pollution in the lake’s surface sediments. Further integrating Monte Carlo simulation techniques, this study incorporates the probability distributions of pollutant concentrations and human exposure parameters into risk assessment models to mitigate uncertainties in the evaluation process. It conducts health risk assessments for different population groups, providing a scientific basis and decision support for the precise remediation of heavy metal pollution in Liuye Lake’s surface sediments, ecological conservation, and the safeguarding of urban residents’ health. Additionally, it provides a representative case study and methodological reference for heavy metal research in sediments of small- to medium-sized urban lakes across China.

## 2. Materials and Methods

### 2.1. Study Area Overview

**Figure 1:**
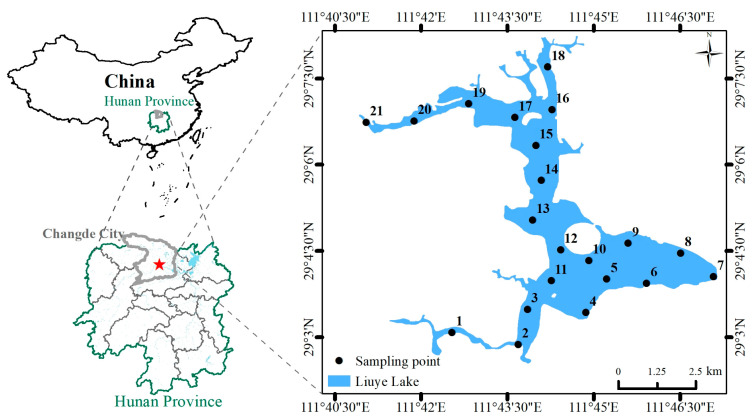
Location of Liuye Lake and Sampling Distribution Map.

Liuye Lake is located in the northeastern part of Changde Ancient City, Hunan Province, China, and is directly adjacent to the central urban area. As a representative urban inland lake, it is simultaneously affected by urban expansion and performs critical functions in regulating the urban microclimate and sustaining regional ecological stability. As illustrated in Figure 1, the lake has an irregular patchy morphology, with a core water area of about 21.8 km^2^, a highly meandering shoreline, and a well-developed aquatic–terrestrial ecotone ecosystem. The mean water depth ranges from 3 to 4 m, with no clear differentiation between shallow and deep zones. The lake possesses moderate hydraulic exchange capacity, and its water quality stably meets the Class III criteria specified in the Environmental Quality Standards for Surface Water (GB 3838-2002) [36]. Major water quality indices fully satisfy the ecological functional demands of an urban landscape lake.

As a key hub within Changde’s integrated river–lake system, Liuye Lake is hydraulically connected to several urban rivers, including the Chuanzhi River and Yinyuan River, and serves as a core component of the city’s “Water Network Connectivity” ecological project. Outflow from the lake discharges into West Dongting Lake, forming a continuous hydrological continuum: “urban lakes–urban rivers–Dongting Lake”. Therefore, the water quality condition of Liuye Lake, as well as the speciation, migration, and transformation of heavy metals in its surface sediments, not only dominate its own ecological stability but also exert a cascading influence on the water quality and ecological security of the entire Dongting Lake basin. This renders the lake a critical study area for ecological protection and environmental management research within the basin. All images in this article were created using ArcGIS (10.8) and Origin (2022).

### 2.2. Sample Collection and Analysis

As a typical urban lake, Liuye Lake is strongly affected by urban domestic inputs and scattered agricultural activities in its surrounding catchment. Pollutant distributions show minor spatial discrepancies between nearshore regions and the open-water area. To satisfy the requirements for subsequent Monte Carlo simulation and ensure representative coverage of diverse functional zones across the lake, 21 sediment sampling sites were established in this study (Figure 1). Based on a sample-size sufficiency evaluation, the number of sampling points was verified to be adequate for characterizing regional pollutant distribution patterns and calibrating key model parameters.

The detailed sampling design is described as follows: Sampling sites 1 and 2 were placed at the primary inflow rivers of Liuye Lake, and site 21 was positioned at the main outflow, in order to precisely identify external pollutant input pathways and internal output characteristics. For the main lake area, a grid-based uniform sampling strategy was adopted. Sampling sites 3–20 were uniformly distributed across various ecological units, including nearshore landscape zones, central deep-water areas, and shallow vegetated regions, thereby ensuring full coverage of the entire lake and reducing spatial representativeness bias.

Surface sediment samples at a depth of 0–10 cm were collected using a Peterson grab sampler. At each sampling site, three replicate samples were collected and homogenized to form a single composite sample. All samples were stored in pre-cleaned, acid-washed, and sealed polyethylene bags. During field sampling, geographic coordinates of each site were recorded with a high-precision RTK-GPS instrument (Trimble Inc., Westminster, CO, USA; accuracy 0.1 m), accompanied by supplementary environmental parameters including water transparency, coverage of aquatic vegetation, and intensity of surrounding human activities.Once transported to the laboratory, sediment samples were manually sorted to remove impurities such as aquatic organism residues, rootlets, gravel, and plastic fragments using stainless steel tweezers. The purified samples were then freeze-dried, ground continuously in an agate mortar to a fine powder without visible particles, and passed through a 100-mesh nylon sieve. The sieved fine powder was collected in clean sample bottles and stored for further analysis.

The sample digestion process employs microwave digestion. Accurately weigh 0.10 g of sieved sediment sample into a PTFE digestion vessel. Sequentially add 6 mL of ultrapure nitric acid (ρ = 1.42 g/cm^3^, trace metal grade, ≥69% purity, Merck, KGaA, Darmstadt, Germany) and 2 mL of ultrapure hydrofluoric acid (ρ = 1.15 g/cm^3^, trace metal grade, ≥40% purity, Merck, Germany). Gently shake the digestion vessel to ensure the sample is thoroughly mixed with the acids. Place the vessel in a fume hood and allow it to stand for 30 min to prevent vigorous reaction. Subsequently, seal the digestion vessel with its lid and place it in a MARS 6 microwave digestion system (CEM Corporation, Matthews, NC, USA). The digestion program was set as follows: the temperature was increased to 180 °C at a ramp rate of 10 °C/min, held for 25 min with a maximum power of 1200 W, and then cooled naturally to room temperature. After digestion, add 1 mL of internal standard solution (containing Rh and Re at 10 μg/L each), dilute to the mark with ultrapure water, mix thoroughly, and store at 4 °C in a light-protected refrigerator. Heavy metal concentrations in the pretreated sediment samples were determined using an iCAP-Q inductively coupled plasma mass spectrometer (iCAP-Q ICP-MS, Thermo Fisher Scientific, Waltham, MA, USA). Before formal detection, systematic performance calibration was conducted for the instrument. The optimized working parameters were set as follows: RF power of 1550 W, nebulizer gas flow rate of 0.85 L/min, auxiliary gas flow rate of 0.8 L/min, cooling gas flow rate of 14 L/min, sampling depth of 8 mm, helium flow rate in the collision cell of 4.5 mL/min, and integration time of 0.1 s. A series of standard working solutions with concentrations of 0.1, 1, 10, 50, and 100 μg/L were prepared using the National Institute of Standards and Technology (NIST) standard reference material SRM 2704 (lake sediment) [37]. Calibration curves were established for each target heavy metal, and all correlation coefficients (R^2^) were greater than 0.9995. Strict quality assurance and quality control (QA/QC) procedures were implemented throughout the analytical process to guarantee the reliability of data. Reagent blank tests were run synchronously with each batch of samples, and all target metals in blank samples were below the method detection limits. One replicate sample was inserted for every 10 analyzed samples, and the relative standard deviation (RSD) of parallel determinations was controlled within 5%. The national standard reference material GBW07310 [38] (coastal marine sediment) was used for method validation. The relative errors (RE) between measured values and certified values for As, Cd, Cr, Cu, Mn, Ni, Pb, and Zn ranged from −3.5% to +3.0%. Recovery tests were also conducted by spiking low, medium, and high levels of mixed standard solutions into sediment matrices, and the recoveries of each element fell within 91.8–106.1%. During instrumental detection, sample solutions were atomized into aerosols and introduced into the plasma torch for high-temperature ionization at about 6000 K. Target ions were extracted through sampling and skimmer cones into the vacuum system and separated by a quadrupole mass analyzer according to their mass-to-charge ratios (m/z): As (75), Cd (111), Cr (52), Cu (63), Hg (202), Mn (55), Ni (60), Pb (208), and Zn (66). The signal intensity of each isotope was recorded by the detector, and the actual content of each heavy metal in Liuye Lake surface sediments was quantified based on the external calibration curve.

### 2.3. Heavy Metal Pollution Assessment Methods

#### 2.3.1. Geo-Accumulation Index Method

The geo-accumulation index (*I_geo_*), developed by Müller, is a classical and widely applied method for assessing heavy metal contamination in aquatic sediments. This method takes into account both anthropogenic inputs and natural geochemical processes such as diagenesis, which allows for an objective and accurate evaluation of pollution degree and source contribution. The calculation formula is expressed as follows [39]:

*I_geo_* = log_2_[C/(1.5C_0_)]

where *I_geo_* refers to the geo-accumulation index of heavy metals; C is the measured concentration of heavy metals in sediment samples (mg/kg); and C_0_ represents the regional soil background value of heavy metals (mg/kg). In this study, the soil background values of the Dongting Lake Basin in Hunan Province were used as the reference baseline [40]. Liuye Lake is located within the Dongting Lake Basin, with similar sediment sources, geological background, and hydrological conditions; thus, the background values of this basin are more representative and reasonable for the present study. A constant of 1.5 was applied to correct the disturbance of environmental background values caused by diagenesis. The pollution intensity classification table for the geo-accumulation index is shown in Table 1.

#### 2.3.2. Enrichment Factor Method

The enrichment factor (*EF)* technique is employed to quantify the accumulation degree of heavy metals in sediments. This method effectively eliminates the interference caused by sediment grain size and mineralogical composition. The calculation formula is presented as follows [41]:

$$\mathrm{EF}=\frac{{\left(C_{i}/C_{r}\right)}_{sample}}{{\left(C_{i}/C_{r}\right)}_{background}}$$

In the equation, ${\left(c_{i}/c_{r}\right)}_{sample}$ and ${\left(c_{i}/c_{r}\right)}_{background}$ represent the measured values and background values (mg/kg) of element i in soil from the Dongting Lake basin, respectively, where $C_{r}\;$ denotes the reference element concentration (mg/kg). Aluminum (Al) was adopted as the reference element in this study, owing to its high abundance in the Earth’s crust, strong chemical stability, low volatility, and weak responsiveness to anthropogenic impacts [42]. The classification criteria of EF contamination levels are summarized in Table 2 below.

#### 2.3.3. Potential Ecological Risk Assessment Method

The potential hazardous impacts of individual heavy metals are closely related to their environmental concentrations and toxicological characteristics. The potential ecological risk index model was applied to evaluate the ecotoxicological effects of heavy metals in the study area. The computational formulas are as follows [43]:

$$C_{f}^{i}\;=\;c_{i}/c_{n}^{i}$$

$$E_{f}^{i}=T_{r}^{i}/C_{f}^{i}$$

$$RI=\overset{n}{\underset{i=1}{\sum }}E_{r}^{i}$$

In the formula, $C_{f}^{i}$ refers to the contamination factor of heavy metal *i*, and $c_{i}$ is its measured content in sediments, $c_{n}^{i}$ stands for the soil background value of element *i* in Hunan Province. $E_{f}^{i}$ and $T_{r}^{i}$ represent the individual potential ecological risk index and the toxic-response factor of heavy metal *i*, respectively. In this work, the toxic-response factors for As, Cd, Cr, Cu, Pb, Zn, Mn, Ni, and Hg were set as 10, 30, 2, 5, 5, 1, 1, 5, and 40, respectively [43]. *RI* is defined as the comprehensive potential ecological risk index of all heavy metals (Table 3).

### 2.4. Health Risk Assessment Methods

In this study, the human health risk assessment model recommended by the United States Environmental Protection Agency (USEPA) was adopted to assess non-carcinogenic and carcinogenic risks for adult males, adult females, and children exposed to sediments in the Liuye Lake wetland [44]. Considering the low exposure probability of heavy metals in sediments via inhalation, only two primary pathways—oral ingestion and dermal contact—were evaluated. The computational formulas are presented below [45]:

$$CDD_{ing}\;=\;\;\frac{c^{k}\times {\mathrm{IR}}_{ing}\times \mathrm{ED}\times \mathrm{EF}\times \mathrm{CF}}{\mathrm{BW}\times \mathrm{AT}}$$

$$CDD_{derm}=\;\frac{c^{k}\times \mathrm{SA}\times \mathrm{AF}\times \mathrm{ABS}\times \mathrm{ED}\times \mathrm{EF}\times \mathrm{CF}}{\mathrm{BW}\times \mathrm{AT}}$$

In the above formulas, $CDD_{ing}$ and $CDD_{derm}$ stand for the average daily exposure doses through oral ingestion and dermal contact (mg/kg·d), respectively. $c^{k}$ is the measured concentration of heavy metal *k* in sediments (mg/kg). ${\mathrm{IR}}_{ing}$ refers to the ingestion rate, EF is the exposure frequency, and ED represents the exposure duration. BW denotes body weight of the exposed population, AT is the average exposure time, AF is the skin adherence factor, SA is the skin exposure area, ABS is the dermal absorption coefficient, and CF is the unit conversion factor.

The non-carcinogenic health risks posed by heavy metals in surface sediments of Liuye Lake for different populations were calculated using the following equation [45]:

$$\mathrm{HI}=\;\sum HQ_{ij}\;=\;\sum \frac{CDD_{ing}}{RfD_{ij}}$$

*HI* represents the total hazard index for non-carcinogenic risk, while $HQ_{ij}$ and $RfD_{ij}$ refer to the hazard quotient and reference dose of heavy metal i through exposure pathway *j*, respectively. When *HI* (or *HQ*) > 1, the heavy metal may present potential non-carcinogenic risks to humans; when *HI* (or *HQ*) ≤ 1, the non-carcinogenic risk is negligible.

The carcinogenic risk caused by heavy metals in surface sediments of Liuye Lake for different populations was calculated using the following formula [45]:

$$\mathrm{TCR}=\;\sum CR_{ij}\;=\;\sum CDD_{ij}\;\times \;SF_{ij}$$

*TCR* refers to the total carcinogenic risk, and $CR_{ij}$ and $SF_{ij}$ represent the carcinogenic risk index and carcinogenic slope factor of heavy metal *i* through exposure pathway *j*, respectively. The non-carcinogenic parameters and carcinogenic slope factors of target heavy metals are summarized in Table 4. When *TCR* (or $CR_{ij}$) < 1 × 10^−6^, the carcinogenic risk of heavy metals in sediments is negligible. When 1 × 10^−6^ ≤ *TCR* (or $CR_{ij}$) < 1 × 10^−4^, the carcinogenic risk is generally acceptable. When *TCR* (or $CR_{ij}$) > 1 × 10^−4^, the carcinogenic risk is considered unacceptably high. The key parameters for health risk assessment of different populations are listed in Table 5.

### 2.5. Monte Carlo Model Construction

Traditional heavy metal pollution assessment methods often employ static, fixed values for toxicity response coefficients, background levels, and risk exposure parameters, leading to potential overestimation or underestimation biases in evaluation results. This study employs Monte Carlo simulation to assess heavy metal pollution, conduct risk analysis, and conduct predictive research. The specific assessment workflow proceeds as follows: First, establish a probability density distribution model. Next, clarify the distribution characteristics of each heavy metal element. Then, initiate the model calculation program and perform extensive random sampling. Finally, conduct a statistical analysis on the obtained simulation data. During simulation operations, the random sampling iteration count is set to 10,000 to ensure the statistical analysis achieves a 95% confidence interval (CI). This approach fully accounts for parameter uncertainties and enhances the reliability of evaluation outcomes.

## 3. Results

### 3.1. Characteristics of Heavy Metal Concentrations in Surface Sediments

#### 3.1.1. Statistical Characteristics of Heavy Metal Concentrations in Surface Sediments

Based on heavy metal detection results from Liuye Lake wetland sediments and Anderson–Darling tests (N), optimal distribution fits varied among metals: Cd and Zn followed beta distributions; Pb and Ni exhibited Weibull distributions; As and Hg showed log-normal distributions; Cr followed triangular distributions; Cu followed uniform distributions; and Mn followed discrete uniform distributions.

The differences in the optimal statistical distributions among heavy metals in the same natural environment can be attributed to their distinct geochemical behaviors, sources, migration pathways, and transformation processes in sediment systems.

Elements controlled primarily by natural geological background and soil-forming processes tend to follow symmetric or near-normal distributions. In contrast, metals influenced by anthropogenic inputs, adsorption–desorption behavior, redox conditions, organic matter complexation, and point-source pollution often show skewed distributions such as log-normal, Weibull, beta, triangular, or uniform distributions.

Moreover, differences in mobility, bioavailability, particle-size distribution, and diagenetic modification further diversify their frequency distributions. Therefore, the observed variation in optimal fitting distributions among different heavy metals reflects the combined effects of natural geological background and anthropogenic activities, as well as their inherent geochemical characteristics in the Liuye Lake wetland sediment system.

The concentrations of heavy metals in the sediments of Liuye Lake Wetland are shown in Table 6. The order of average concentrations of each heavy metal in sediments is: Mn (1112.95 mg/kg) > Zn (111.67 mg/kg) > Cr (73.71 mg/kg) > Ni (35.76 mg/kg) > Cu (29.63 mg/kg) > Pb (31.98 mg/kg) > As (12.43 mg/kg) > Cd (0.46 mg/kg) > Hg (0.17 mg/kg). Compared to the background values of the Dongting Lake sediment water system, the average heavy metal concentrations at the Liuye Lake wetland sampling points were all higher than these background values. Specifically, the average concentrations of Hg, Mn, Ni, Cr, Cu, Pb, Cd, Zn, and As were 3.62, 2.47, 1.68, 1.67, 1.47, 1.37, 1.39, 1.34, and 0.96 times higher than their respective background values, respectively. However, localized exceedances of As concentrations were still observed. Regarding the coefficient of variation, Hg exhibited the highest at 55.07%, followed by As at 38.82%. Cd showed a coefficient of 34.43%, Mn at 33.34%, Ni at 20.74%, Pb at 18.59%, Cu at 17.69%, Zn at 17.09%, and Cr with the lowest coefficient of variation at 12.19%.

These results demonstrate that most heavy metals in Liuye Lake surface sediments show elevated average concentrations relative to background values. Among them, Hg displays the most significant enrichment, followed by Mn, while other elements exhibit varying degrees of accumulation. Only As shows a mean concentration slightly lower than the background baseline, yet localized hotspots with elevated As levels still exist across the lake. Spatial heterogeneity of heavy metal contents differs substantially among elements. Hg has the largest coefficient of variation and the most uneven spatial pattern, suggesting potential local anthropogenic anomalies. As, Cd, and Mn also exhibit relatively high spatial variability and poor distribution uniformity. In contrast, Ni, Pb, Cu, and Zn show lower variability, with Cr being the lowest. These elements are distributed more uniformly and are less affected by point-source pollution.

#### 3.1.2. Spatial Distribution Characteristics of Heavy Metals in Surface Sediments

**Figure 2:**
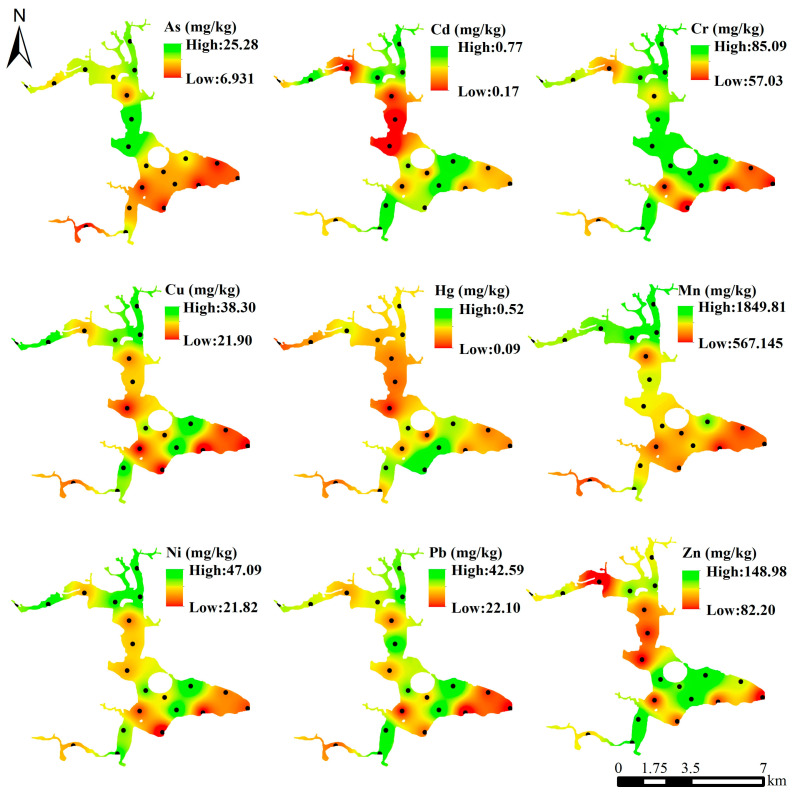
Spatial distribution of heavy metal concentrations in surface sediments of Liuye Lake.

The concentrations of nine heavy metals (As, Cd, Cr, Cu, Hg, Mn, Ni, Pb, Zn) in the surface sediments of Liuye Lake show significant spatial variations (Figure 2). The distribution of their high and low concentration zones is closely associated with the lake’s hydrogeological connectivity, the spatial distribution of surrounding human activities, and the rules of material transport and deposition, which is consistent with the spatial variability analysis results of heavy metal concentration statistics.

Cd concentrations ranged from 0.17 to 0.77 mg/kg and Cr from 57.03 to 85.09 mg/kg, with their high-concentration zones concentrated at the main inflow estuaries of urban rivers and densely human-distributed nearshore areas. These areas receive continuous exogenous pollutants from urban domestic sewage and surface runoff; the reduced water flow velocity causes heavy metals to settle rapidly with sediments, making sediments the primary carrier for Cd and Cr accumulation. In contrast, the lake interior, far from pollution sources, has significantly lower heavy metal deposition due to water dilution and long-distance transport, leading to low concentrations.

As concentrations varied from 6.93 to 25.28 mg/kg, with its high-concentration zones scattered—besides the main inflows, small-scale enrichment was observed in local shallow-water vegetation areas, corresponding to its localized over-standard characteristics. This is attributed to As input by surface runoff from soil erosion and agricultural non-point source pollution, and the particulate adsorption by aquatic vegetation that exacerbates As sedimentation and accumulation. In the well-mixed deep waters of the lake center, As concentrations remained generally low.

Cu, Ni, Pb and Zn presented relatively uniform spatial distributions with gentle high-low concentration gradients: Cu (21.90–38.90 mg/kg), Ni (21.82–47.09 mg/kg), Pb (22.10–42.59 mg/kg) and Zn (82.20–148.98 mg/kg). Slightly high concentrations of these four metals were only found at a few Lakeside areasuch as agricultural irrigation ditch estuaries and urban green belts, with no obvious regional enrichment. This aligns with their statistical features of low variation coefficients and weak susceptibility to point-source pollution, as their distributions are mainly controlled by the regional natural geological background, with only minor localized superimposed impacts from human activities.

Hg and Mn showed distinct spatial distribution patterns from other heavy metals and had the strongest spatial variability. Hg concentrations ranged from 0.09 to 0.52 mg/kg, with patchy high-concentration zones distributed in the traffic-dense northwestern nearshore areas and around small domestic sewage outlets, while concentrations in other areas were relatively low. Local abnormally high Hg values resulted in a 55.07% variation coefficient, reflecting the significant impact of specific anthropogenic point-source emissions and distinct spatial heterogeneity of Hg pollution input. Mn had the widest concentration range (567.145–1849.81 mg/kg) among all tested elements, with high-concentration zones concentrated at both inflow and outflow estuaries: Mn carried by rivers settled rapidly with sediments at inflows, while intense water-sediment interface interactions during flood discharge and water exchange promoted Mn migration from water to sediments at outflows. Thus, Mn concentrations at inflows and outflows were significantly higher than in other lake areas, with relatively uniform concentrations in the lake interior.

Overall, heavy metals in the surface sediments of Liuye Lake exhibited a core spatial pattern: slightly higher nearshore concentrations, lower lake center concentrations, enrichment at inflows, and localized anomalies. Moreover, the element distribution was highly correlated with their pollution sources: Cd, Cr and Hg, dominated by anthropogenic exogenous inputs, showed greater spatial heterogeneity with high-concentration zones matching densely human-activity areas; Cu, Ni, Pb and Zn, controlled by natural geological factors, had relatively uniform distributions; Mn, affected by both natural sources and hydrological processes, presented typical dual enrichment at inflows and outflows; As, influenced by natural background and local non-point source pollution, had scattered high-concentration zones. These spatial distribution characteristics provide an intuitive spatial basis for subsequent identification of heavy metal pollution sources and key polluted areas in Liuye Lake.

### 3.2. Characteristics of Heavy Metal Pollution in Surface Sediments

#### 3.2.1. Geo-Accumulation Index

**Figure 3:**
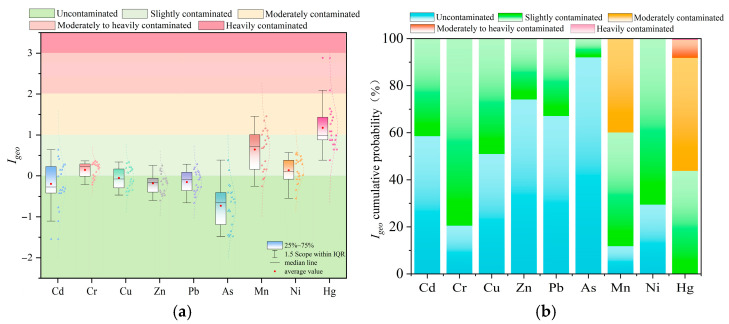
Assessment results of the geo-accumulation index and corresponding probability: (**a**) Geo-accumulation index evaluation; (**b**) contamination probability of the geo-accumulation index simulated by the Monte Carlo method.

Based on the calculation of the geo-accumulation index, the mean *I_geo_* values of heavy metals in the surface sediments of Liuye Lake are determined as follows: Hg (1.11) > Mn (0.64) > Cr (0.15) > Ni (0.14) > Cu (−0.05) > Pb (−0.15) > Zn (−0.18) > Cd (−0.20) > As (−0.74). According to the *I_geo_* pollution grading standards, Hg indicates moderately polluted levels, Mn, Cr, and Ni indicate slightly polluted levels, while Cu, Pb, Zn, Cd, and As indicate no pollution. The Monte Carlo simulation-based *I_geo_* prediction averages for sediment heavy metals are: Hg (1.18) > Mn (0.74) > Cr (0.15) > Ni (0.13) > Cu (−0.03) > Pb (−0.15) > Zn (−0.19) > Cd (−0.20) > As (−0.73). Combined with the Monte Carlo simulated pollution probability distribution in Figure 3b, Hg exhibits a 48.01% probability of moderate contamination and a 43.79% probability of mild contamination. Mn shows probabilities of 39.90% for moderate contamination, 48.30% for mild contamination, and 11.80% for no contamination, indicating overall mild contamination but with potential risk of escalation to moderate contamination. Cr, Ni, Cu, Pb, Zn, Cd, and As predominantly exhibit mild contamination or no contamination, with combined probabilities exceeding 50% for both states and near-zero probabilities for moderate or higher contamination. This is consistent with the assessment results obtained from the geo-accumulation index.

#### 3.2.2. Enrichment Factors

**Figure 4:**
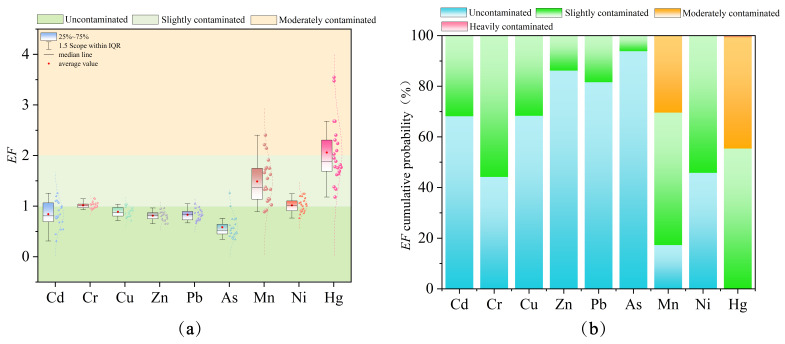
Evaluation results of enrichment factors and corresponding probabilities: (**a**) enrichment factor assessment; (**b**) enrichment contamination probability simulated by the Monte Carlo method.

According to the enrichment factor calculation, the mean *EF* values of heavy metals in the surface sediments of Liuye Lake are as follows: Hg (2.06) > Mn (1.49) > Cr (1.02) = Ni (1.02) > Cu (0.89) > Cd (0.84) > Pb (0.83) > Zn (0.81) > As (0.58). According to the *EF* pollution grading standards, Hg is moderately polluted, while Mn, Cr, and Ni are slightly polluted; Cu, Pb, Zn, Cd, and As show no pollution. The Monte Carlo simulation-based average EF predictions for sediment heavy metals are: Hg (2.08) > Mn (1.63) > Ni (1.03) > Cr (1.02) > Cu (0.91) > Cd (0.86) > Pb (0.84) > Zn (0.82) > As (0.59). As shown in Figure 4b, the probability of moderate Hg contamination is 44.01%, while that of mild contamination is 55.23%. For Mn, the probabilities are 30.4% for moderate contamination, 52.32% for mild contamination, and 17.28% for no contamination. Cr, Ni, Cu, Pb, Zn, Cd, and As predominantly exhibit mild contamination or no contamination, with combined probabilities for these two states exceeding 50% and probabilities for moderate or higher contamination approaching zero. This aligns with the enrichment factor evaluation results.

Based on the integrated geo-accumulation index (*I_geo_*), measured enrichment factors (*EF*), and Monte Carlo simulation results, the surface sediments of Liuye Lake are predominantly mildly polluted by heavy metals. Hg exhibits relatively higher pollution levels, while Mn poses a risk of escalating pollution. The pollution risks for other elements are low. The Monte Carlo simulation results generally align with the measured data, showing only minor local variations.

**Figure 5:**
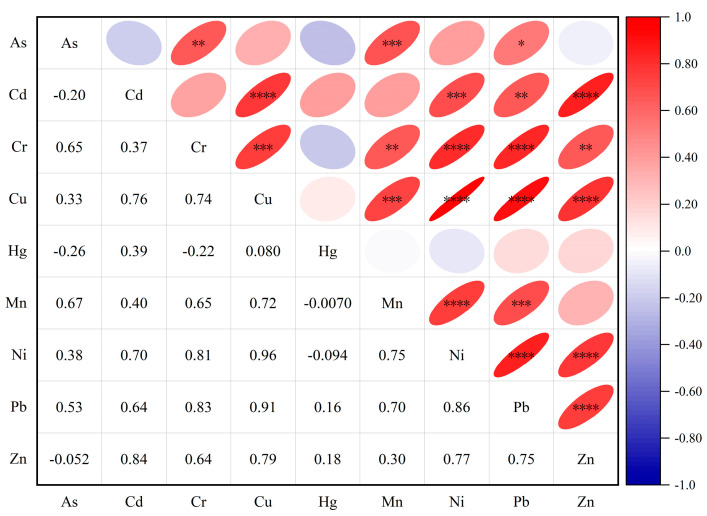
Pearson correlation coefficients for heavy metal concentrations in surface sediments of Liuye Lake. Note: * indicates *p* < 0.05, ** indicates *p* < 0.01, *** indicates *p* < 0.001, and **** indicates *p* < 0.0001; the shade of the color bars indicates the magnitude of the correlation coefficient. An elliptical shape sloping upward indicates a positive correlation, while one sloping downward indicates a negative correlation; the flatter the ellipse, the stronger the correlation; the rounder the ellipse, the weaker the correlation.

As shown in Figure 5. To further clarify the sources of heavy metal pollution, a Pearson correlation analysis was conducted on the concentrations of nine heavy metals. The results indicate that Cu, Pb, Zn, and Cd exhibit a highly significant positive correlation (*p < 0.001) with high correlation coefficients, suggesting that these four elements share similar environmental behavior and anthropogenic sources. Cr and Ni show a highly significant positive correlation (*p < 0.001); their bioaccumulation factors and pollution levels are similar, reflecting a common natural geological background. Hg showed no significant correlation with any other heavy metals, indicating a relatively independent source primarily controlled by specific anthropogenic point-source emissions; Mn exhibited weak correlations with most heavy metals, but its enrichment factor was relatively high, and the probability of moderate contamination reached 30.4%, demonstrating a composite characteristic of natural geochemical background superimposed on anthropogenic inputs from the watershed; As showed weak correlations with all elements and had a low enrichment factor, primarily dominated by natural sources. Combining the enrichment factor analysis with the correlation analysis reveals that heavy metals in the surface sediments of Liuye Lake primarily originate from natural geological sources. Cu, Pb, Zn, and Cd are influenced by mixed anthropogenic sources such as transportation, agriculture, and domestic sewage; Hg represents typical anthropogenic point-source pollution; and Mn exhibits composite pollution from both natural and anthropogenic sources, along with a potential risk of escalation. These findings are consistent with the previous evaluation of enrichment factors and the results of the Monte Carlo simulation.

Slight discrepancies exist between *I_geo_* and *EF* evaluations because *EF* normalizes data using a reference element (Al) to eliminate grain size effects, whereas *I_geo_* does not apply grain size correction [47]. Monte Carlo simulation was used to calculate the 95% confidence intervals of *I_geo_* and *EF*, which can effectively quantify the uncertainty of pollution assessment. The deterministic calculation results are generally consistent with the simulated mean values, but the probabilistic results can further show the probability distribution of pollution levels. The differences between deterministic and probabilistic results are relatively small, indicating that the pollution assessment results are highly reliable. Hg and Mn show relatively larger uncertainty, suggesting potential pollution escalation risks. Monte Carlo simulation quantifies data uncertainty and projects pollution trends, addressing the limitation of measured data reflecting only current conditions. Integrating both approaches achieves complementary advantages, accurately depicting the pollution landscape and providing scientific support for pollution prevention, control, and risk prediction.

### 3.3. Assessment of Potential Ecological Risks from Heavy Metals in Sediments

**Figure 6:**
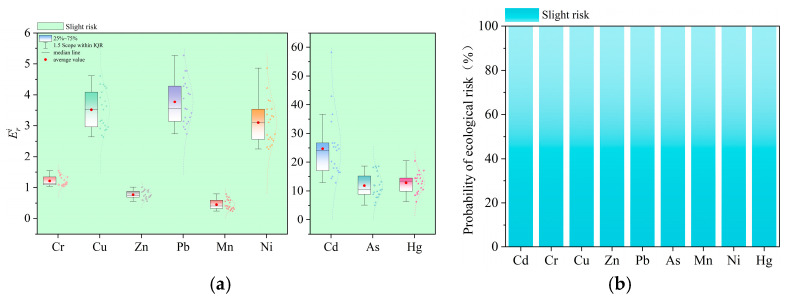
Comprehensive potential ecological risk assessment results and corresponding probability: (**a**) integrated potential ecological risk evaluation; (**b**) comprehensive potential ecological risk probability simulated by the Monte Carlo method.

Calculations of potential risk values indicate that the average $E_{f}^{i}$ values for heavy metal elements in the surface sediments of Liuye Lake are: Cd (24.72) > Hg (12.80) > As (11.83) > Pb (3.77) > Cu (3.51) > Ni (3.10) > Cr (1.21) > Zn (0.77) > Mn (0.45). All heavy metals exhibit mild pollution levels. The average *RI* value is 62.71, indicating a low ecological risk level. The average *EF* values for sediment heavy metals predicted by Monte Carlo simulation are: Cd (24.71) > Hg (12.83) > As (11.85) > Pb (3.76) > Cu (3.45) > Ni (2.96) > Cr (1.17) > Zn (0.76) > Mn (0.37), with an average *RI* of 61.86. As shown in Figure 6b, all heavy metal elements exhibit mild pollution levels, consistent with the aforementioned evaluation results.

**Figure 7:**
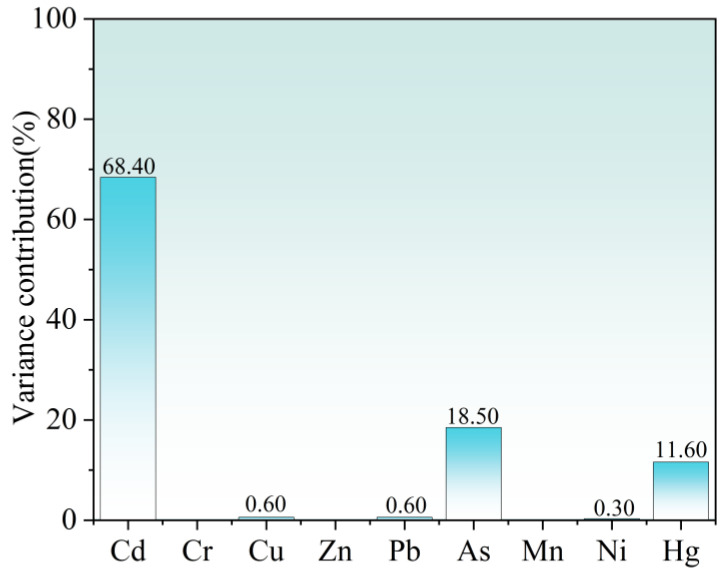
Contribution of Each Heavy Metal to the Comprehensive Potential Ecological Risk.

To further quantify the contribution of each heavy metal to the overall ecological risk, sensitivity analysis was carried out in this study. As shown in Figure 7, The results demonstrate that Cd, Hg, and As are the primary contributors to the potential ecological risk posed by heavy metals in Liuye Lake surface sediments. This can be attributed to the fact that the potential ecological risk index integrates the toxic-response factors of heavy metals, and the toxicity coefficients of Cd, Hg, and As are notably higher than those of other elements, leading to their dominant roles in risk contribution. Although the overall contamination level of surface sediments in Liuye Lake is relatively low, long-term environmental monitoring and targeted risk control are still necessary due to the high toxicities and potential ecological hazards of Cd, Hg, and As. Special attention should be paid to identifying the pollution sources and migration mechanisms of these three elements, so as to safeguard the long-term health and stability of the regional aquatic ecosystem.

The 95% confidence intervals of the potential ecological risk index (*RI*) obtained by Monte Carlo simulation further verify the uncertainty of ecological risk assessment. The deterministic RI values are highly consistent with the probabilistic simulation results, and the overall uncertainty is low, which fully confirms that the surface sediments of Liuye Lake are at slight ecological risk with high credibility.

Furthermore, the combination of Monte Carlo simulation and comprehensive potential ecological risk assessment not only strengthens the reliability and robustness of analytical outcomes, but also effectively overcomes the inherent limitations of conventional potential ecological risk assessment methods, especially the shortage of quantitative analysis on the contribution rates of individual heavy metal elements.

### 3.4. Human Health Risk Assessment

In the human health risk assessment, average physiological parameters for adult males, adult females, and children were used, including average daily exposure doses via hand-mouth ingestion and dermal contact, ingestion rate, exposure frequency, exposure duration, body weight, average exposure time, skin adherence factor, skin exposure surface area, and dermal absorption factor. All parameters were derived from USEPA exposure guidelines and relevant population exposure handbooks, with clear and authoritative supporting bases.

**Figure 8:**
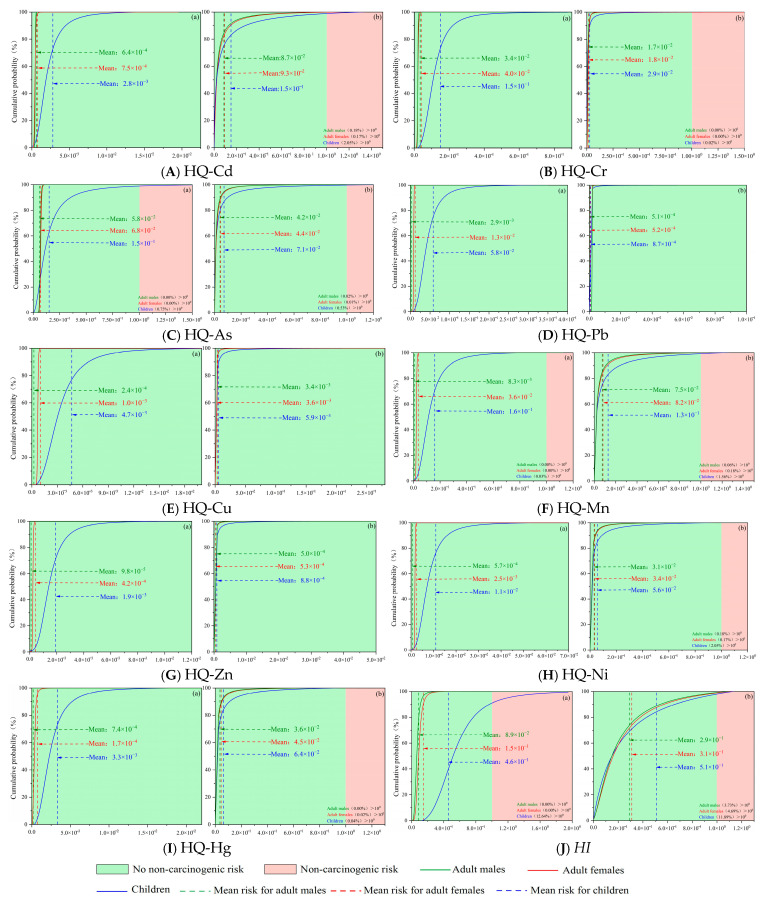
Non-carcinogenic health risk probability assessment for single heavy metals and total heavy metals. (**a**) hand-mouth ingestion; (**b**) dermal contact.

Figure 8 depicts the cumulative probability curves corresponding to health risks induced by sediment-borne heavy metals for three distinct population groups, namely adult males, adult females and children. Regarding non-carcinogenic risks via oral ingestion pathway, no adverse risks were detected for all population groups exposed to Cd, Cr, Pb, Cu, Zn, Ni and Hg. Specifically, arsenic (As) posed no non-carcinogenic risk to adult males and adult females, yet carried a 0.75% probability of triggering non-carcinogenic risk for children. Mn exhibited no non-carcinogenic risk for adult males and females, with a merely 0.03% risk probability for children. For oral exposure to individual heavy metals, the descending order of average hazard quotient (*HQ*) values across different populations was ranked as: As > Cr > Mn > Pb > Hg > Cd > Ni > Cu > Zn.

With respect to dermal contact exposure, no non-carcinogenic risk was observed for all population groups upon exposure to Pb, Cu and Zn. For adult males, the non-carcinogenic risk probabilities of Cd, Cr, As, Mn, Ni and Hg were 0.18%, 0.00%, 0.02%, 0.06%, 0.18% and 0.00%, respectively; for adult females, the corresponding risk probabilities were 0.17%, 0.00%, 0.01%, 0.18%, 0.17% and 0.02%, respectively; for children, the values reached 2.05%, 0.02%, 2.05%, 1.56%, 2.05% and 0.04%, respectively. The descending order of average *HQ* values for individual heavy metals across different populations via dermal exposure was: Cd > Mn > As > Hg > Ni > Cr > Cu > Pb > Zn. These findings demonstrate that the hazard quotient values of heavy metals vary noticeably among different population groups, and such discrepancies are closely dependent on exposure pathways.

For heavy metal exposure via hand-to-mouth contact, the comprehensive non-carcinogenic risk indices (*HI*) for adult males, adult females, and children were 0.15, 0.31, and 0.66, respectively—all below the risk threshold of 1. However, children exhibited a 12.64% probability of exceeding an *HI* value of 1. For dermal exposure, the *HI* values for the three groups were 0.29, 0.31, and 0.50, respectively. The probabilities of exceeding the threshold were 3.73%, 4.69%, and 11.89% for adult males, adult females, and children, respectively. Both exposure routes present non-carcinogenic risks, though the overall risk levels remain low. Notably, children exhibit the highest non-carcinogenic risk across both exposure pathways. This disparity is primarily attributed to factors such as weaker metabolic capacity, relatively higher exposure doses, distinct behavioral patterns, and greater heavy metal absorption rates in children [48].

**Figure 9:**
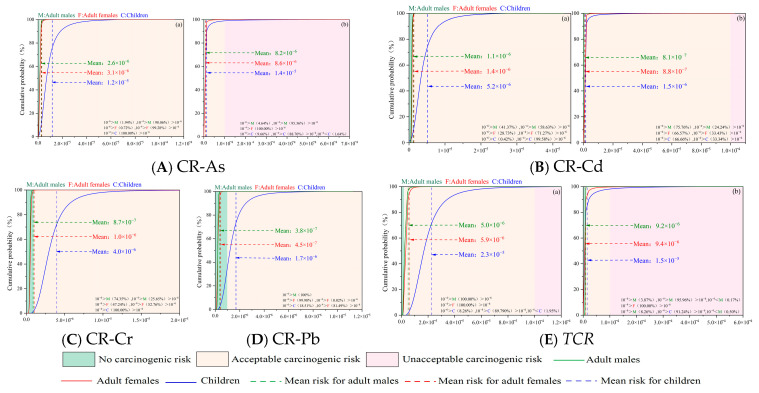
Carcinogenic health risk probability assessment for single heavy metals and total heavy metals. (**a**) hand-mouth ingestion; (**b**) dermal contact.

Figure 9 illustrates the cumulative probability curves of carcinogenic risks induced by sediment-borne heavy metals for distinct population groups (adult males, adult females, and children). The computed carcinogenic risk (CR) values of Cd, Cr, and Pb via diverse exposure pathways for all target groups were all below the acceptable risk threshold (CR < 10^−4^), indicating no unacceptable carcinogenic hazards. For the hand-to-mouth oral ingestion pathway, arsenic (As) presented non-carcinogenic risk probabilities of 1.94%, 0.72%, and 0.00% for adult males, adult females, and children, respectively, while the corresponding acceptable carcinogenic risk probabilities reached 98.06%, 99.28%, and 100.00%. For dermal contact exposure, As exhibited negligible carcinogenic risk probabilities of 4.64%, 0.00%, and 9.66% for the three groups, in sequence, with acceptable risk probabilities of 95.36%, 100.00%, and 88.70%; notably, the probability of As exceeding the carcinogenic risk threshold for children was 1.64%. Regarding Cd exposure via the hand-to-mouth pathway, the negligible carcinogenic risk probabilities were 41.37%, 28.73%, and 0.42% for adult males, adult females, and children, respectively, and the acceptable risk probabilities were 58.63%, 71.27%, and 99.58%. For dermal contact with Cd, the negligible carcinogenic risk probabilities were 75.76%, 66.57%, and 66.66%, while the acceptable risk probabilities were 24.24%, 33.43%, and 33.34% for the three groups, respectively. Constrained by data availability and model applicability, this study only calculated the carcinogenic risks of Cr and Pb via the hand-to-mouth exposure pathway. For Cr, the negligible carcinogenic risk probabilities were 74.35%, 47.24%, and 0.00% for adult males, adult females, and children, respectively, with corresponding acceptable risk probabilities of 25.65%, 52.76%, and 100.00%. For Pb, the negligible carcinogenic risk probabilities reached 100.00%, 99.98%, and 18.51%, and the acceptable risk probabilities were 0.00%, 0.02%, and 81.49% for the three groups, in turn. Monte Carlo simulation characterizes the uncertainty of non-carcinogenic and carcinogenic risks through probability distribution and 95% confidence intervals. The differences between deterministic and probabilistic results are mainly reflected in the small probability of exceeding the risk threshold, especially for children. The probabilistic results can more comprehensively support health risk early warning.

The average TCR values for heavy metals in Liuye Lake surface sediments across different groups indicate that, overall, the total carcinogenic risk from heavy metals remains within acceptable limits for all populations. However, children face an unacceptable high carcinogenic risk via oral ingestion (1.95%) and skin contact (0.50%). This aligns with non-carcinogenic risk assessment results, primarily due to children’s lower resistance to toxic substances.

It should be noted that this study employed the USEPA-recommended health risk assessment model for Liuye Lake surface sediments, with parameters referenced from USEPA’s general standards. These may deviate from the actual environmental conditions in the Liuye Lake area, introducing certain limitations to this research. Concurrently, health risks from heavy metal exposure via hand-to-mouth ingestion and skin contact are closely tied to individual characteristics such as gender, age, and occupational type among exposed populations. The primary population in the Liuye Lake area consists of local urban residents. For this group, more refined methods are required to calculate heavy metal exposure doses. Consequently, future research should further explore and optimize the evaluation system and methodology for assessing the health risks of heavy metals in Liuye Lake’s surface sediments.

Compared with studies on other small and medium-sized urban lakes, the overall health risk of heavy metals in surface sediments of Liuye Lake is relatively low, and both non-carcinogenic and carcinogenic risks are within acceptable ranges, which is consistent with typical urban lakes such as East Lake (Wuhan, China) [49] and Xuanwu Lake (Nanjing, China) [50].

Meanwhile, this study also found that the potential health risk for children is significantly higher than that for adults, with As and Cr as the main contributing elements. This pattern is common in urban lakes, indicating that children’s health is more sensitive to urban lake sediments.

Different from urban lakes strongly affected by industry and heavy traffic, Liuye Lake is mainly influenced by domestic sources, agricultural non-point sources and natural geological background without obvious industrial pollution, resulting in lower overall health risks, which conforms to the environmental characteristics of small and medium-sized urban lakes.

## 4. Discussion

### 4.1. Investigation into the Causes of Heavy Metal Pollution in Surface Sediments of Liuye Lake

#### 4.1.1. Impact of Human Activities on Heavy Metal Pollution in Sediments

In urban lake ecosystems, anthropogenic activities act as the dominant driving force for heavy metal enrichment and accumulation in surface sediments. Unlike remote lakes governed primarily by natural background values, urban lakes suffer from more direct and intense disturbances derived from surrounding residential and production activities. Integrated with the foregoing pollution risk assessment results, heavy metal contamination in Liuye Lake sediments is mainly driven by anthropogenic sources including domestic sewage discharge from adjacent towns, urban traffic abrasion, atmospheric deposition, and scattered industrial emissions, with marked discrepancies in contribution profiles among different heavy metals, which conforms to the universal pollution pattern of urban shallow lakes [51]. Urban domestic sewage represents a critical input pathway of heavy metals into the lake. Untreated direct domestic sewage discharge and combined rain–sewage runoff in towns surrounding Liuye Lake continuously carry Hg and Cd originating from household commodities and daily chemical products into the water column, which eventually settle and accumulate in sediments. Meanwhile, urban surface runoff scours road surfaces and residential areas, transporting sediment, domestic waste, and surface-borne pollutants, and continuously delivers Cr, Ni and other heavy metals into the lake. This finding is highly consistent with the assessment results of mild Cr and Ni contamination and moderate Hg contamination in this study, and aligns with the pollution source characteristics of similar urban lakes [52]. Additionally, pesticides and fertilizers applied in urban landscaping maintenance are leached and drained into the lake via rainfall, further exacerbating the secondary enrichment of certain heavy metals and elevating pollution risks in local zones.

Urban traffic emissions and wastewater and waste gas from scattered surrounding small-scale industries serve as core triggers for the intensification of specific heavy metal pollution. Hg and Pb generated from vehicle exhaust, tire wear, and brake system abrasion enter the lake directly through two pathways: dry and wet atmospheric deposition and surface runoff, constituting vital anthropogenic sources of Hg and Pb contamination in the lake. Despite the absence of large-scale industrial discharge, the cumulative effect of long-term disorderly discharge of Hg- and Mn-containing production wastewater from nearby small processing facilities has directly led to the highest Hg pollution level among all heavy metals in Liuye Lake, while Mn also presents mild contamination with a continuous upward risk trend. Simultaneously, Cr and Ni particles produced by road surface abrasion are carried into the lake by rain runoff and enriched in sediments, which is fully consistent with the urban geographical feature of a dense traffic network and large traffic flow around Liuye Lake [53].

#### 4.1.2. Influence of Natural Causes on Sediment Heavy Metal Pollution

In addition to anthropogenic disturbances, natural geological background and hydrological sedimentary conditions act as fundamental controlling factors for heavy metal accumulation in Liuye Lake sediments, determining the environmental background levels of heavy metals. The lithology of the Liuye Lake Basin is rich in rock-forming elements including Mn, Cr, and Ni [54]. Long-term physical and chemical weathering of regional bedrock and watershed soil erosion continuously release these natural elements, which are transported to the lake via surface runoff and deposited in sediments, forming the natural background accumulation of heavy metals—this is the core mechanism underlying the natural sources of certain heavy metals. The enrichment factors of Mn, Cr, and Ni in this study are close to 1, directly indicating that the accumulation of these three heavy metals is primarily controlled by natural geological processes, with relatively weak superimposed disturbances from human activities, classifying them as natural background-dominated elements. This result is also highly consistent with the regional geological and geochemical characteristics [55]. Meanwhile, the low background content of As in the regional geology corresponds to the absence of As contamination in the study results, further verifying the fundamental regulatory effect of natural background on the heavy metal pollution pattern.

Hydrological dynamics and sedimentary conditions are key natural factors regulating the migration, transformation, and enrichment of heavy metals. As a typical urban shallow lake, Liuye Lake features weak hydrodynamic conditions, a long water exchange cycle, and poor water fluidity. Such hydrological traits greatly slow the migration and diffusion rate of heavy metal particles, providing favorable conditions for their sedimentation and accumulation, and making it difficult for heavy metals to be exported via water exchange, leading to long-term retention in sediments. Furthermore, sediment grain size and organic matter content are core physicochemical factors affecting heavy metal adsorption and immobilization. Fine-grained sediments have a large specific surface area, and organic matter exhibits strong complexation and adsorption capacity for heavy metals. Thus, areas with abundant fine particles and high organic matter content show stronger adsorption and fixation of highly toxic heavy metals such as Hg and Cd, resulting in a spatial distribution pattern of local moderate to severe Hg contamination [56], which is fully consistent with the characteristics of local high-value Hg pollution zones identified by the foregoing spatial analysis. From a critical perspective, although natural factors serve as the basic control for heavy metal accumulation, their contribution to the overall pollution pattern is significantly lower than that of anthropogenic sources under high-intensity urban human disturbances, which represents the core difference in pollution driving mechanisms between urban lakes and natural lakes.

### 4.2. Primary Factors Affecting Human Health in the Liuye Lake Region

**Figure 10:**
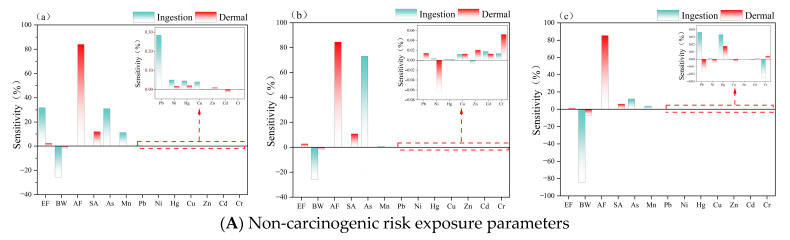

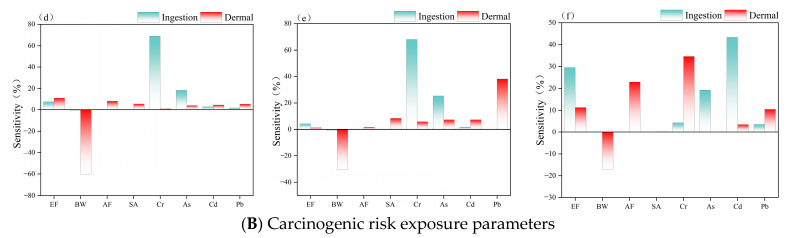
Sensitivity maps for probabilistic assessment of non-carcinogenic and carcinogenic health risks: (**a**,**d**) adult males; (**b**,**e**) adult females; (**c**,**f**) children.

Based on the preceding non-carcinogenic and carcinogenic probabilistic health risk assessment results, combined with parameter sensitivity analysis via Monte Carlo simulation, this section further identifies the core sensitive parameters and key driving factors of the health risk assessment model, clarifies the contribution differences of different heavy metals, exposure pathways, and population physiological characteristics to health risks, addresses the limitations of purely numerical risk assessment, and enhances the scientificity and pertinence of the research conclusions. As shown in Figure 10, in non-carcinogenic health risk assessments for different populations, the contribution rates of arsenic (As) and manganese (Mn) are markedly higher than those of other heavy metals, with all other heavy metals contributing less than 1%. This result stands in clear contrast to the finding that Cd and Hg are the core risk factors in ecological risk assessment, reflecting the fundamental logical differences between ecological risk and human health risk assessment systems, which is also one of the key findings of this study.

Regarding the influence of human physiological indicators on non-carcinogenic health risks: for the hand-to-mouth ingestion pathway, the sensitivity of body weight (BW) is −25.65% for adult males, −25.85% for adult females, and −84.39% for children, showing a significant negative correlation. Compared with adults, BW shows extremely high sensitivity for children, indicating that greater body weight correlates with stronger resistance to toxicant exposure. Children’s lower body weight and underdeveloped metabolic system make them a high-risk group for non-carcinogenic health risks around the lake, requiring targeted enhanced protection measures, which is consistent with the foregoing health risk assessment findings. Notably, children have a higher frequency of outdoor activities, and their probability of hand-to-mouth contact with sediment and soil in wetland and tidal flat areas is far higher than that of adults, further increasing heavy metal intake via oral exposure and elevating health risks—this is the core behavioral reason for children’s significantly higher risk level than adults. For dermal exposure, the sensitivity of the skin adhesion factor (AF) is 83.98% for adult males, 84.50% for adult females, and 85.35% for children. All age groups show extremely high AF sensitivity with minimal intergroup differences, suggesting that pollutant adhesion characteristics on the skin surface are a critical factor in dermal exposure risk, and dermal exposure traits are consistent across populations. This finding carries important methodological implications, indicating that future similar studies should refine measured AF parameters to replace default model values, further improving the accuracy of health risk assessments and reducing uncertainty from model parameters.

In carcinogenic health risk assessments across different populations, exposure pathways associated with chromium (Cr) and arsenic (As) are far more sensitive than those of other heavy metals. This discrepancy with ecological risk sensitivity analysis stems from the different focuses of the two assessment systems: ecological risk assessment integrates heavy metal environmental concentrations and ecological toxicity coefficients, and Cd and Hg rank highest in toxicity among the nine heavy metals, thus dominating ecological risk sensitivity. In contrast, human health risk assessment prioritizes the carcinogenic and pathogenic effects of heavy metals on humans. Although As and Cr occur at lower environmental concentrations, they are classified as potent carcinogens, and long-term low-dose exposure can pose significant carcinogenic risks to humans [57], resulting in a high contribution rate in health risk evaluations. Consistent with non-carcinogenic sensitivity results, for the hand-to-mouth pathway, exposure frequency (EF) has high explanatory power for adult carcinogenic risk, and carcinogenic risk is negatively correlated with BW: higher body weight corresponds to lower carcinogenic risk. For children, both exposure pathways show high sensitivity for both physiological metrics and heavy metal elements, significantly exceeding adult levels. This aligns with previous health risk assessment findings, mainly attributed to children’s weaker metabolic capacity, higher heavy metal absorption rate, and immature body defense system. From a critical standpoint, the sensitivity analysis in this study identifies core health risk control factors and reveals parameter limitations of existing health risk models. Future research can optimize model parameters combined with field monitoring data and develop dedicated risk prevention protocols for children to improve public health security around urban lakes [58].

## 5. Conclusions

This study took surface sediments from Liuye Lake in Hunan Province as the research object, and determined the concentrations of nine heavy metal elements, namely Hg, Mn, Cr, Ni, Cu, Pb, Zn, Cd and As. A series of comprehensive evaluation methods were adopted, including the geo-accumulation index (*I_geo_*), enrichment factor (EF), potential ecological risk assessment and human health risk assessment. Furthermore, Monte Carlo simulation was integrated to quantify the data uncertainty and evolutionary trends of pollution levels. On this basis, the content distribution characteristics, pollution status, potential ecological hazards and human health risk probabilities of heavy metals in surface sediments of Liuye Lake were systematically analyzed. The main findings and conclusions are summarized as follows:

(1) The average concentrations of the nine heavy metals in the surface sediments of Liuye Lake follow the descending order: Mn > Zn > Cr > Ni > Pb > Cu > As > Cd > Hg. Compared with the background values of the Dongting Lake sediment system, the average concentrations of eight heavy metals are all higher than the corresponding background thresholds, with the exception of As. Among these elements, Hg and Mn show significant over-standard enrichment, while the remaining heavy metals exhibit varying degrees of accumulation relative to the background level. Despite the average As concentration being slightly lower than the background value, localized point exceedances of As are still observed in the study area. The spatial variability of heavy metal concentrations presents remarkable inter-element differences. Hg possesses the highest coefficient of variation, suggesting the existence of potential local concentration anomalies and discontinuous spatial distribution. As, Cd, and Mn also show relatively high coefficients of variation, representing poor uniformity and stability in their spatial distribution patterns. In contrast, Ni, Pb, Cu, Zn, and Cr display lower coefficients of variation, indicating relatively uniform spatial distribution and weaker susceptibility to anthropogenic point-source pollution in the study region.

(2) Based on measured geo-accumulation indices (*I_geo_*) and enrichment factors (*EF*) alongside Monte Carlo simulation results, surface sediments in Liuye Lake exhibit overall mild contamination, with varying degrees across elements. Both *I_geo_* and EF assessments indicate Hg as the most heavily contaminated, followed by Mn, Cr, and Ni, showing mild contamination. Cu, Pb, Zn, Cd, and As were found to be uncontaminated overall. Monte Carlo simulations indicate a potential risk of Mn escalating to moderate contamination, while other elements show no significant escalation trends. Simulation results generally align with measured data, with only minor local variations.

(3) Potential ecological risk assessment results demonstrate that the descending order of average single heavy metal potential ecological risk values ($E_{f}^{i}$) in Liuye Lake surface sediments is: Cd > Hg > As > Pb > Cu > Ni > Cr > Zn > Mn. All target heavy metals are classified at the mild ecological risk level, and the average comprehensive potential ecological risk index (*RI*) is 62.71, suggesting that the overall ecological risk of the study area is at a mild degree. Furthermore, sensitivity analysis identifies Cd, Hg and As as the core dominant elements contributing to the total ecological risk, which is closely associated with their relatively high toxic-response coefficients.

(4) Human health risk assessment coupled with Monte Carlo simulation illustrates that different heavy metals pose differentiated health risks to various population groups via diverse exposure pathways. For non-carcinogenic risks, the total non-carcinogenic risks via oral ingestion and dermal contact pathways are generally low, yet children suffer from noticeably higher non-carcinogenic hazards than adult groups. Specifically, the probability of children exceeding the threshold of the comprehensive non-carcinogenic risk index (*HI*) is 12.64% for oral ingestion and 11.89% for dermal contact, respectively. As and Mn are verified as the primary contributing elements to non-carcinogenic health risks.

With respect to carcinogenic risks, the overall carcinogenic risk levels under different exposure scenarios are within the internationally acceptable range. Nevertheless, children face an extremely low probability of encountering unacceptable carcinogenic risks. Cr and As are the core contributors to carcinogenic health risks, which is consistent with the strong inherent carcinogenicity of the two heavy metal elements.

## Figures and Tables

**Table 1:** *I_geo_* contamination level classification criteria [39].

Level	Pollution Index	Pollution Category
0	*I_geo_* ≤ 0	Uncontaminated
1	0 < *I_geo_* ≤ 1	Slightly contaminated
2	1 < *I_geo_* ≤ 2	Moderately contaminated
3	2 < *I_geo_* ≤ 3	Moderately to heavily contaminated
4	3 < *I_geo_* ≤ 4	Heavily contaminated
5	*I_geo_* > 4	Extremely contaminated

**Table 2:** *EF* contamination level classification criteria [41].

Level	Pollution Index	Pollution Category
0	*EF* ≤ 1	Uncontaminated
1	1 < *EF* ≤ 2	Slightly contaminated
2	2 < *EF* ≤ 5	Moderately contaminated
3	5 < *EF* ≤ 20	Moderately/Heavily contaminated
4	20 < *EF* ≤ 40	Heavily contaminated
5	*EF* > 40	Extremely contaminated

**Table 3:** Classification criteria for the comprehensive potential ecological risk index (*RI*) [43].

Level	Pollution Index	Risk Class
0	*RI* ≤ 150	Slight risk
1	150 < *RI* ≤ 300	Moderate risk
2	300 < *RI* ≤ 600	Higher risk
3	600 < *RI* ≤ 1200	High risk
5	*RI* > 1200	Extremely high risk

**Table 4:** Reference Dose (*RfD*) and Slope Factor (*SF*) for Heavy Metals in Health Risk Assessment [45].

Elements	*RfD*		*SF*	
	Ingestion	Dermal	Ingestion	Dermal
As	3.00 × 10^−4^	1.23 × 10^−4^	1.50 × 10^0^	1.50 × 10^0^
Cd	1.00 × 10^−3^	1.00 × 10^−5^	1.80 × 10^0^	3.80 × 10^−1^
Cr	3.00 × 10^−3^	6.00 × 10^−5^	5.00 × 10^−1^	-
Cu	4.00 × 10^−2^	1.20 × 10^−2^	-	-
Hg	3.00 × 10^−4^	2.10 × 10^−5^	-	-
Ni	2.00 × 10^−2^	5.40 × 10^−3^	-	-
Pb	3.50 × 10^−3^	5.25 × 10^−3^	8.50 × 10^−3^	-
Zn	3.50 × 10^−1^	6.00 × 10^−2^	-	-
Mn	4.60 × 10^−2^	1.84 × 10^−3^	-	-

**Table 5:** Probability Parameter Distribution for Health Risk Assessment of Heavy Metals in Sediments [46].

Exposure Parameters	Unit	Probability Distribution	Adult Males	Adult Females	Children
IRing	mg/d	point	114	114	200
ED	a	point	70	70	18
EF	d/a	triangular	345 (180–365)	345 (180–365)	345 (180–365)
BW	kg	logarithmic	67.55 ± 8.72	57.59 ± 8.03	-
BW	kg	triangular	-	-	29.30 (5.20–56.80)
ABS	-	point	0.03 (As), 0.14 (Cd), 0.001 (Cr), 0.1 (Cu), 0.35 (Ni), 0.006 (Pb), 0.02 (Zn), 0.01 (Mn), 0.50 (Hg)		
SA	m^2^	triangular	0.169 (0.085–0.422)	0.153 (0.076–0.382)	0.086 (0.043–0.216)
AF	mg/cm^2^·d	logarithmic	0.49 ± 0.54	0.49 ± 0.54	0.65 ± 1.2
CF	-	point	10 (−6)	10 (−6)	10 (−6)
AT (non-carcinogenic)	d	point	365 × ED	365 × ED	365 × ED
AT (carcinogenic)	d	point	365 × 70	365 × 70	365 × 70

Triangular distribution: most probable value (minimum, maximum); logarithmic distribution: mean ± standard deviation.

**Table 6:** Heavy Metal Concentrations in Sediments of Liuye Lake Wetland.

	Cd	Cr	Cu	Zn	Pb	As	Mn	Hg	Ni
Min/(mg/kg)	0.17	57.00	21.90	82.20	22.10	6.93	567.00	0.09	21.80
Max/(mg/kg)	0.77	85.10	38.30	149.00	42.60	25.30	1850.00	0.30	47.10
Median/(mg/kg)	0.41	77.70	28.70	112.00	32.80	12.30	1106.00	0.14	34.10
Mean/(mg/kg)	0.46	73.71	29.63	111.67	31.98	12.43	1112.95	0.17	35.76
SD	0.16	8.98	5.24	19.08	5.95	4.82	371.08	0.10	7.42
CV/%	34.43	12.19	17.69	17.09	18.59	38.82	33.34	55.07	20.74
Background value (Dongting Lake)/(mg/kg)	0.33	44.00	20.20	83.30	23.30	12.90	450.00	0.047	21.20
TF	30	2	5	1	5	10	1	40	5
Distribution type	Beta	triangular	uniform	Beta	Weibull	Log-normal	Discrete uniform	Log-normal	Weibull

## Data Availability

The original contributions presented in this study are included in the article. Further inquiries can be directed to the corresponding author.

## Abbreviations

The following abbreviations are used in this manuscript:

USEPA	United States Environmental Protection Agency
SD	Standard Deviation
CV	Coefficient of Variation
TF	Toxicity factor
